# Alteration of tumor suppressors changes the endometrial tumor spectrum

**DOI:** 10.15252/emmm.202318166

**Published:** 2023-08-17

**Authors:** Lindsey D Mayo

**Affiliations:** ^1^ Herman B Wells Center for Pediatric Research, Department of Pediatrics, Indiana University School of Medicine Indiana University Indianapolis Indianapolis IN USA

**Keywords:** Cancer, Urogenital System

## Abstract

The most common gynecological cancer in Europe and the United States is endometrial. Like most cancers, early‐stage endometrial cancer has a more favorable prognosis, while high‐grade, including endometrioid and nonendometrioid, has the worst prognosis. In endometrioid human tumors, the tumor suppressor genes PTEN and p53 (Trp53) are frequently altered or lost, as identified in datasets from The Cancer Genome Atlas. These suppressors' somatic mutations or loss of gene expression can lead to neoplastic development, tumor progression, and therapeutic resistance. In addition, somatic missense mutations are prevalent in another tumor suppressor, the F‐box and WD repeats containing 7 (FBXW7). FBXW7 is part of the SCF‐βTrCP ubiquitin complex that signals protein destruction. Specifically, FBXW7 is responsible for binding and facilitating the destabilization of proteins involved in proliferation and migration. Losing the function of multiple tumor suppressors could activate pathways involved in neoplastic progression, malignancy, therapeutic resistance, and formation of different tumor subtypes. The study by Brown *et al* in this issue of *EMBO Mol Med* (Brown *et al*, 2023) provides insight into the complexity of tumor suppressor mutations in malignant endometrial cancer.

## Graphical abstract

L. Mayo discusses the study by Brown *et al*, in this issue of *EMBO Mol Med*, that uses genetically engineered mouse models to determine the functional consequences of missense and loss of function mutations in Fbxw7, Pten, and Tp53 in the context of high‐risk endometrial cancers.

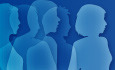

The authors studied endometrial tumor development in genetically engineered mouse models. They examined the pathological characteristics generated by uterus‐driven Cre recombination of *Fbxw7* ^*K482Q*^ *(human Fbxw7* ^*K479Q*^ *)*, *Trp53* deletion, *Fbxw7* ^*K482Q*^ *Trp53* deletion, gain of function *Trp53* ^*R1*72H^ (human mutant *p53* ^*R175H*^), *Fbxw7* ^*K482Q*^ *Trp53* ^*R1*72H^, *deletion pten* ^−/−^, and *Fbxw7* ^*K482Q*^ *pten* ^*−/−*^. Mice carrying *Fbxw7* ^*K482Q*^ alone did not develop endometrial cancer, while those with *pten* deletion did. Interestingly, mice with *Trp53* ^*−/−*^ mutation did not develop endometrial tumors, but soft tissue sarcomas at 24 weeks. Similarly, mice carrying p53 hotspot mutant *Trp53* ^*R1*72H^ showed early onset tumor development outside the uterus; however, a small percentage exhibited endometrial carcinomas. Complex genetic recombination showed that mice with *pten* ^*−*/−^ *Fbxw7* ^*K482Q*^ developed high‐grade endometrial tumors as early as 6 weeks. Interestingly, the presence of both *p53* ^*R1*72H^ and *Fbxw7* ^*K482Q*^ also accelerated endometrial tumor formation.

Differential gene expression revealed an elevated Wnt signaling pathway in the *Fbxw7* ^*K482Q*^ *pten* ^−/−^ tumors compared with the *pten* ^*−/−*^ tumors. This observation was confirmed by an analysis from the TCGA. A virtual (*in silico*) screen identified several proteins from the Wnt pathway, including LEF (Lymphoid enhancer element) and TCF7L2 (Transcription factor 7 like‐2), as potentially able to bind FBXW7. Molecular approaches verified that LEF1 and TCF7L2 bound to the WD40 domain of FBXW7. LEF was detected by immunohistochemistry in *Fbxw7* ^*K482Q*^ mouse endometrial tumors, and its presence was further increased in *pten* ^*−/−*^ or *Trp53* ^*R1*72H^ co‐mutant animals. Thus, the findings by Brown *et al* suggest an upregulation of the Wnt pathway during endometrial tumor progression.

Interestingly, these mouse models shared some features with human endometrial cancer. Indeed, the invasive nature of cells undergoing EMT requires a network of transcription factors and cellular signaling. The Wnt pathway is known to induce the EMT transcription factor, Snai2 (Saegusa *et al*, 2009; Kobayashi & Ozawa, 2013), and it would thus be expected that mutations in FBXW7 alone would be sufficient to drive tumor formation; however, this study revealed that endometrial cancers were highly penetrant only when PTEN was deleted (Daikoku *et al*, 2008). Loss of PTEN can cause the dysregulation of numerous pathways, including AKT and FAK‐Rac (Tamura *et al*, 1998; Maehama & Dixon, 1999), both involved in tumor migration via Snai‐mediated regulation of E‐cadherin (Sun *et al*, 2021) and Fak‐Rac regulation of lamellipodin and cell migration, respectively. Activating these pathways would contribute to the EMT program (Brabletz *et al*, 2018). Thus, combining FBXW7 mutation and PTEN loss would dramatically increase Wnt signaling, kinase signaling pathways, and EMT transcription factors. The addition of missense mutation to p53 could also contribute to this program, as observed in a small fraction of *pten* ^*−/−*^ *Fbxw7* ^*K482Q*^ animals.

An important aspect of the study by Brown *et al* is to reveal the different impacts of a specific missense mutant compared with the loss of p53 on endometrial tumor development. Similarly, specific catalytic domain knock‐in mutants of PTEN versus knockout mice were shown to lead to a diverse tumor spectrum (Wang *et al*, 2010). These findings highlight the difference between the loss of a gene and a missense mutation with a gain of function.

While some pathways were common between humans and mice, caution must be exercised as differences have been described in the normal function of the p53‐targeted genes between species. Specific missense mutations in p53 and the EMT program are also different. Mouse models suggest that *Fbxw7* ^*K482Q*^ is not a driver of endometrial neoplasms. Instead, mutant p53 or loss of PTEN appear to be drivers, and Fbxw7^K482Q^ accelerates endometrial tumor development. It is noteworthy that mouse cells require far fewer genetic alterations for malignant transformation than human cells. Brown *et al* report a good correlation between the mouse model, human cells, and genetic alterations. It will be interesting to assess whether human endometrial cells require more/less genetic alterations in specific pathways, including Wnt; whether specific mutants of FBXW7 are better drivers of tumor formation; or whether other pathways like TGFβ, EGFR, or hypoxia are contributors to tumor development, malignancy, or resistance. The current study thus provides a foundation to test additional hypotheses on the different events necessary for the development of endometrial tumors and/or the formation of tumor subtypes and identifies some key elements, that is, FBXW7, Wnt, PTEN, and Trp53.

## Author contribution

**Lindsey Mayo:** Conceptualization.

## References

[emmm202318166-bib-0001] Brabletz T , Kalluri R , Nieto MA , Weinberg RA (2018) EMT in cancer. Nat Rev Cancer 18: 128–134 29326430 10.1038/nrc.2017.118

[emmm202318166-bib-0002] Brown M , Leon A , Kedzierska K , Moore C , Belnoue‐Davis HL , Flach S , Lydon JP , DeMayo FJ , Lewis A , Bosse T *et al* (2023) Functional analysis reveals driver cooperativity and novel mechanisms in endometrial carcinogenesis. EMBO Mol Med 10.15252/emmm.202217094 PMC10565641 37589076

[emmm202318166-bib-0003] Daikoku T , Hirota Y , Tranguch S , Joshi AR , DeMayo FJ , Lydon JP , Ellenson LH , Dey SK (2008) Conditional loss of uterine Pten unfailingly and rapidly induces endometrial cancer in mice. Cancer Res 68: 5619–5627 18632614 10.1158/0008-5472.CAN-08-1274 PMC2824329

[emmm202318166-bib-0004] Kobayashi W , Ozawa M (2013) The transcription factor LEF‐1 induces an epithelial‐mesenchymal transition in MDCK cells independent of β‐catenin. Biochem Biophys Res Commun 442: 133–138 24269234 10.1016/j.bbrc.2013.11.031

[emmm202318166-bib-0005] Maehama T , Dixon JE (1999) PTEN: a tumour suppressor that functions as a phospholipid phosphatase. Trends Cell Biol 9: 125–128 10203785 10.1016/s0962-8924(99)01519-6

[emmm202318166-bib-0006] Saegusa M , Hashimura M , Kuwata T , Okayasu I (2009) Requirement of the Akt/beta‐catenin pathway for uterine carcinosarcoma genesis, modulating E‐cadherin expression through the transactivation of slug. Am J Pathol 174: 2107–2115 19389926 10.2353/ajpath.2009.081018 PMC2684176

[emmm202318166-bib-0007] Sun J , Chen W , Wen B , Zhang M , Sun H , Yang X , Zhao W , La L , An H , Pang J *et al* (2021) Biejiajian pill inhibits carcinogenesis and metastasis via the Akt/GSK‐3β/snail signaling pathway in hepatocellular carcinoma. Front Pharmacol 12: 610158 33762939 10.3389/fphar.2021.610158 PMC7982731

[emmm202318166-bib-0008] Tamura M , Gu J , Matsumoto K , Aota S , Parsons R , Yamada KM (1998) Inhibition of cell migration, spreading, and focal adhesions by tumor suppressor PTEN. Science 280: 1614–1617 9616126 10.1126/science.280.5369.1614

[emmm202318166-bib-0009] Wang H , Karikomi M , Naidu S , Rajmohan R , Caserta E , Chen HZ , Rawahneh M , Moffitt J , Stephens JA , Fernandez SA *et al* (2010) Allele‐specific tumor spectrum in pten knockin mice. Proc Natl Acad Sci U S A 107: 5142–5147 20194734 10.1073/pnas.0912524107 PMC2841921

